# Investigating the causes of brain ischaemia: value of arterial spin labelling for perfusion imaging

**DOI:** 10.1007/s00330-025-12175-y

**Published:** 2025-11-21

**Authors:** Wilhelm Kuker

**Affiliations:** Wolfson Centre for the Prevention of Stroke and Dementia, Nuffield Department of Clinical Neurology, University of Oxford, Oxford, UK

Ischaemic stroke is a major cause of death and disability. The treatment of acute brain vessel occlusions has made significant progress in recent years [1, 2]. However, stroke prevention by treating the underlying risk factors remains important to reduce overall morbidity from brain ischaemia.

One of the main causes of stroke is stenoses and wall irregularities of the brain-supplying arteries due to atheromatous plaques. Extensive clinical trials established the relation between the severity of the stenoses and the stroke risk. They also proved the benefit of early carotid revascularisation by carotid endarterectomy (CEA) in symptomatic patients [3]. Two large prospective trials found significant treatment benefit for patients with carotid stenoses exceeding 70%. Initially, formal angiography was used to measure the degree of stenosis. This was then replaced by non-invasive CTA, MRA, and Doppler ultrasound. The latter allows to assess changes in blood flow velocity to quantify the carotid stenosis.

Luminal imaging can be complemented by the measurement of brain perfusion.

Arterial spin labelling (ASL) is a well-established MRI method to map the blood supply of the brain.

In this issue of *European Radiology*, Carrozzi et al [4] publish a comprehensive meta-analysis of the literature relating to brain perfusion changes before and after the treatment of carotid stenoses.

They identified 20 studies with over 700 patients who were investigated with ASL before and after either carotid endarterectomy or stenting. The published data showed overall improvement of brain perfusion after treatment of the underlying carotid stenoses.

Patients with large areas of underperfused brain were more likely to suffer from brain haemorrhage post-treatment (reperfusion haemorrhage). However, it remained unclear in a third of patients whether they had been symptomatic from their stenoses before. Also, perfusion changes were not correlated with the degree of the carotid stenoses, possible further stenoses of brain-supplying arteries or the function of the circle of Willis.

Nevertheless, this meta-analysis confirms the utility of perfusion imaging to identify haemodynamic strokes, assess the risk of asymptomatic stenoses [5] and to predict the occurrence of reperfusion haemorrhage after revascularisation. It predicts the improvement of neurological deficits caused by hypoperfusion before permanent structural damage has occurred. It also shows the lack of improvement in patients with completed infarcts.

However, most strokes are caused by thromboembolic events, selectively occluding certain brain arteries, not by reducing overall brain perfusion. This is not intuitive, as the risk of stroke increases with the degree of the carotid stenosis.

Only stenoses exceeding 70% combined with an incomplete circle of Willis [6] cause an overall reduction of brain perfusion.

Until then, the stenoses are compensated for by accelerated blood flow (Bernoulli effect). In addition, there is the recruitment of collateral blood supply from branches of the external carotid artery.

Instead, high-grade carotid stenoses cause blood flow turbulence with subsequent clot formation. They are also associated with large areas of endothelial damage and an increasing amount of necrotic plaque content, which might become embolic material.

Embolic and haemodynamic strokes differ in semiology and imaging characteristics [7]. The former are usually distinct, sometimes recurrent events, often causing ischaemic lesions in the cortex or adjacent white matter. The perfusion deficits are confined to the supply area of the occluded brain vessel.

Typical haemodynamic infarcts (border zone infarcts) can be found in the depth of the hemispheric white matter (internal border zones, “string of pearls”) or the periphery of the supply areas of the large brain arteries (“watershed infarcts”). Neurological deficits usually fluctuate with blood pressure and may be reversible for an extended period of time [7]. These infarcts are associated with larger areas of reduced brain perfusion, which often do not progress to infarction.

Current evidence only supports the invasive treatment of high-grade and symptomatic carotid stenoses [8, 9].

There is the remaining problem of identifying patients outside these criteria who may benefit from either carotid artery stenting (CAS) or CEA. This applies to symptomatic patients with stenoses of less than 70% and asymptomatic patients with severe carotid stenoses.

Carotid wall imaging can identify high-risk plaque features [10]. A large lipid core demonstrated with MRI or a hypoechoic smooth plaque seen in ultrasound portend an increased incidence of embolic events. Plaque contrast enhancement as a sign of inflammatory changes is also associated with a higher stroke risk.

However, these plaque characteristics are not part of common stroke prediction tools such as the widely used “Oxford carotid stenosis tool” (https://app.calconic.com/public/calculator/5ebab413626c5d00292a7c31?layouts=true).

In clinical practice, the prediction tools are based on demographic and medical factors [11], which are easier (and cheaper) to obtain. High-resolution plaque imaging is time-consuming and, in most institutions, not routinely performed.

Larger studies seem warranted to establish the role of plaque imaging in the management of carotid stenoses, including its cost-effectiveness.

Symptomatic intracranial atherosclerotic stenoses are more prevalent in Asia than in Europe. However, even here, the incidence is increasing in an ageing population [12].

Stenoses of the intracranial arteries are more difficult to detect and quantify than proximal carotid stenoses.

Intracranial time-of-flight MRA at 3 T and CTA are valuable imaging methods for the large intracranial brain-supplying arteries. However, quantification of the intracranial stenoses may be difficult, and perfusion mapping can help to establish their haemodynamic significance.

This has become more relevant as there is increasing evidence of the benefit of endovascular treatment in selected intracranial stenoses. With this new equipoise, further research using advanced imaging methods such as perfusion and vessel wall imaging seems warranted to establish the best management of high-grade intracranial stenoses.

In conclusion, the authors give an excellent overview of the utility of perfusion mapping, particularly ASL, to the imaging workup of patients with suspected ischaemic symptoms. If added to an imaging protocol encompassing MRA and structural brain imaging with DWI, perfusion mapping can identify areas of hypoperfusion before structural brain damage occurs. Restoring brain perfusion by treating high-grade stenoses can prevent symptom progression and reverse transient neurological deficits caused by haemodynamic impairment. Adding perfusion imaging may be particularly useful in the workup of symptomatic patients without high-grade proximal carotid artery stenoses, and further research should focus on this patient group.

ASL, instead of a contrast-based method, might be particularly useful in patients with contraindication to contrast and who undergo time-of-flight angiography instead of contrast-enhanced MRA.

Otherwise, contrast-based MRI or CT perfusion imaging could be preferable.
